# Matching trial design decisions to the needs of those you hope will use the results: the PRECIS-2 tool

**DOI:** 10.1186/1745-6215-16-S2-P223

**Published:** 2015-11-16

**Authors:** Kirsty Loudon, Merrick Zwarenstein, Frank Sullivan, Peter Donnan, Shaun Treweek

**Affiliations:** 1 University of Aberdeen, Aberdeen, UK; 2 University of Dundee, Dundee, UK; 3 Western University, London, Canada; 4 University of Toronto, Toronto, Canada

## 

Randomised trials are hard work. Like much that is hard, this toil is only worth it because of the prospect of a substantial reward. Sadly, the reward to potential users such as patients, healthcare professionals and policy makers is often smaller than it should be because trial design decisions reduced the relevance of the trial to them.

PRECIS-2 is a tool designed to help trialists match their design decisions to the information needs of those they hope will use the trial results. It is an update of the 2009 PRECIS tool, which though highly cited had some well-known weaknesses. PRECIS-2 was developed in collaboration with over 80 international trialists, methodologists and others to produce a tool that addressed those weaknesses but also supports improved design insight for trialists.

PRECIS-2 retains the wheel format of the original tool. It has nine design domains including Eligibility, Recruitment, Setting and Primary outcome. A new domain - Organisation - is explicitly aimed at making trialists consider the resource requirements their intervention will place on health care systems if it were to be rolled out into routine care, the intention being to think about implementation at the design stage. The highly visual presentation makes inconsistent decision-making immediately obvious; it also highlights differences of opinion between trial team members.

We will present the tool, explain how to use it and show examples of how it has been used already. This work is part of the Trial Forge initiative to improve trial efficiency.

